# 

**Published:** 1824-11

**Authors:** 


### MONTHLY CATALOGUE OF MEDICAL BOOKS.

*** *It having been hinted to us that gentlemen, sending copies of their Works, prefer having their titles given at length, it is our intention, in future, to comply with this suggestion: but it is to be observed, that no books can be entered on this List except those sent to us for the purpose;—finding, in the lists hitherto transmitted, that the names of works have frequently been given as published, which have not appeared for weeks, or even months after.*

Lectures on Digestion and Diet. By CHARLES TURNER THACKRAH, Member of the Royal College of Surgeons of London; of the Societé de Medecine pratique de Paris, &c.—Pp. 156. London, 1824.

An Inquiry into the Mode in which Mercurial Ptyalism operates in the Cure of acute Disease; and whether there exist other Remedies, of similar Action, that may be advantageously substituted for it. By JAMES ARNOTT, Author of a Work on Diseases of the Urinary Organs.—Pp. 16. London, 1824.

The Anatomy of the Brain, adapted for the use of Students; comprising Directions with regard to the Method to be pursued in its Dissection, conformably to the Mode practised by most Anatomists.—Pp. 152. London, 1824.

Commentaries on Diseases of the Stomach and Bowels of Children. By ROBLEY DUNGLISON, M.D.; Lecturer on Midwifery and the Diseases of Women and Children; Member of the Royal Academy of Marseilles; of the Royal Society of Sciences, Arts, Belles Lettres, and Agriculture, of Nancy; of the Society of the Faculty of Physicians, the Pharmaceutical and Linnean Societies, of Paris; the Physico-Medical Society of Erlangen; the Academic Medical Society of Marseilles; Secretary for Foreign Correspondence to the Medical Society, and Member of the Hunterian Society, of London; Consulting Accoucheur to the Eastern Dispensary, &c. &c.—Pp. 201. London, 1824.

Hints towards the Adoption of an improved Principle of Remunerating the Surgeon-Apothecary, or General Practitioner in Medicine. Addressed to the Members of the Profession and the Public at large. By T. M. GREENHOW, Member of the Royal College of Surgeons in London; Surgeon to the Lying-in Hospital, to the Charity for poor Married Women lying-in at their own Houses, and to the Infirmary for Diseases of the Eye, Newcastle.—8vo. pp. 26. Newcastle, 1824.

### NOTICE TO CORRESPONDENTS.

*Mr. W. may rest assured, that he is not* the practitioner in Bath, *to whom we alluded in the Review of* Mr. CALVERT'S *Work.*

*We regret that* Sir L. MACLEAN'S *Paper did not arrive in time for the present Number; it shall be given in the next.*

